# Strychnine in Cough Mixtures

**Published:** 1878-05

**Authors:** 


### EXCERPTA.

STRYCHNIA IN COUGH MIXTURES.—Dr. J. Milner Fothergill (London letter, Philadelphia *Medical Times*, Jan. 19, 1878), concludes that of all the agents which exercise a stimulant effect upon the nervous mechanism of respiration, strychnia is one of the most potent and useful. He says:

"Strychnia acts powerfully upon the expiratory part of the respiratory act, and kills by producing spasms of the muscles connected with expiration. It is very useful, then, when expiratory efforts are required for the expulsion of mucus gathered in the air tubes. In chronic bronchitis, with emphysema, it is of great service, and in the dyspnœa connected with advanced Bright's disease it is very efficacious. It produces good effects when given alone, and is a useful addition to ordinary cough mixtures. A combination of carbonate of ammonium, tincture of nux vomica, and tincture of squills, is a most excellent mixture for patients suffering from dyspnœa, and generally procures them "more breath," as they phrase it. One of the most important matters connected with such use of strychnia is its relation to sleep. In many of these cases sleeplessness is a prominent factor; and sleep can be procured only by a narcotic. But while the narcotic acts upon the nervous system generally, it also acts upon the respiration, probably at its center in the medulla, and the patients are apt to wake up with an attack of dyspnœa. A series of cases has demonstrated that by the use of strychnia the respiration is so improved that the patient can go to sleep without the narcotic, and, more than that, sleep fairly well, and be quite free from attacks of breathlessness, which awaken the patient and cause him to add voluntary respiratory efforts to the automatic acts of respiration. By resort to strychine these patients can be much relieved. * * * * By the use of strychnia during the day, a narcotic pill at bed-time is often deprived of its tendency to produce nocturnal dyspnœa; and strychnia may be usefully prescribed in cases of shortness of breath, where there has been also long indulgence in hypnotics. There is no such thing in this world as unalloyed good, and strychnia, so used, sometimes acts so powerfully upon the bladder-centers, and produces such irritation there, as to necessitate its discontinuance. But this is not the rule by any means."

